# Resilience and mental health in university students post-COVID-19 pandemic: insights from the Republic of Cyprus

**DOI:** 10.3389/fpubh.2025.1638427

**Published:** 2025-09-01

**Authors:** Natasja Kudzai Magorokosho, Alexandros Heraclides, Eleonora Papaleontiou-Louca, Maria Prodromou

**Affiliations:** ^1^School of Sciences, European University Cyprus, Nicosia, Cyprus; ^2^School of Humanities, Social & Education Science, European University Cyprus, Nicosia, Cyprus

**Keywords:** COVID-19 pandemic era, university students, mental health, COVID-19, post-pandemic, public health

## Abstract

**Background:**

The COVID-19 pandemic profoundly affected higher education globally. While the immediate psychological effects of the pandemic are well-documented, the long-term impacts on mental health and the potential moderating role of resilience among this population, remain understudied.

**Methods:**

This cross-sectional study aimed to explore the complex interplay between the impact of the COVID-19 pandemic, resilience, and the multifaceted mental health outcomes experienced by university students in the Republic of Cyprus during the post-pandemic era. Participants were from the 4 major districts (Nicosia, Limassol, Larnaca, Paphos) and data was collected between April–July 2024. Three validated scales were used to assess the overall impact of the COVID-19 pandemic (Covid Impact Scale; CIS), mental health symptoms (Symptom Checklist 90-Revised; SCL-90-R), and resilience (Connor-Davidson Resilience Scale; CDRISC), among a sample of 1,017 students. Linear regression analysis was conducted to determine the associations among COVID-19 impact, resilience and mental health.

**Results:**

Higher COVID-19 Impact was associated with poorer mental health (*β* = 2.54, *p* < 0.001). Higher resilience was associated with lower COVID-19 Impact (*β* = −0.08, *p* < 0.001). Moderation analysis revealed that among students with high resilience, COVID-19 Impact was more strongly associated with worse mental health (*β* = 3.09, *p* < 0.001) compared to students with low resilience (*β* = 1.52, *p* < 0.001). Resilience was significantly associated with mental health (*β* = −0.31, *p* < 0.001).

**Conclusion:**

The COVID-19 pandemic had a significant and persistent negative impact on the mental health of Cypriot university students, even after the end of the pandemic, which was profound even among students with high resilience. Further research is needed to better understand the long-term implications of major health crises and to identify effective interventions to promote resilience and mental well-being.

## Introduction

The COVID-19 pandemic profoundly disrupted higher education worldwide, threatening students’ academic progress and psychological well-being (1–3). University students experienced significant hardships, including the abrupt transition to online learning, financial instability, and curtailed social interaction (4, 5). While the immediate psychological effects of the pandemic are well-documented (4, 6, 7), long-term impacts on mental health outcomes and the potential protective role of resilience remain understudied, especially in the post-pandemic era. This introduction provides a contextual overview of these key variables; mental health outcomes, COVID-19 impact, and resilience and outlines the rationale for the present study.

The mental health of university students has been a growing concern throughout the pandemic. Studies have reported elevated levels of anxiety, depression, stress, and loneliness, driven by academic uncertainty, reduced access to support services, and social isolation (8–10). The Republic of Cyprus, characterized by its relatively small and cohesive population (923.381), encountered disruptions intensified by structural and systemic issues within its higher education system (11–13). National estimates report that approximately 17.2% of the population experience mental health issues, with particularly high rates of anxiety (7.2%), depression (3.8%), and substance use disorders (2.6%) among young adults (14). By concentrating on districts within the Republic’s jurisdiction, this study ensures direct applicability to Cyprus’s national public health strategy, university-level services, and policymaking infrastructure (15, 16). The students’ mental health vulnerabilities were exacerbated by these challenges, underscoring the need for ongoing monitoring and support (14, 17, 18). Understanding how mental health outcomes have evolved during the post-pandemic era is essential for informing interventions that promote student well-being.

This study focuses exclusively on the four fully government-controlled districts of the Republic of Cyprus; Nicosia, Limassol, Larnaca, and Paphos. This geographical delineation ensures methodological consistency and alignment with the national health system, as mental health care in these areas is centrally administered by the Ministry of Health under the General Healthcare System (GHS) (19). Furthermore, these districts represent the country’s primary academic centers, hosting a concentration of public and private universities that serve the majority of the student population. There are a total of 8 universities, these include European University Cyprus, Neapolis University Pafos, University of Nicosia, Cyprus University of Technology, University of Central Lancashire University of Cyprus, Frederick University and The Open University which were accredited during data collection period. The Open University of Cyprus (OUC) was excluded due to its distinct distance-learning model and older, part-time student population, which differs markedly from the full-time, demographic targeted in this study. Including OUC could have introduced variability inconsistent with the study’s methodological focus.

The impact of COVID-19 on university students in Cyprus follows global patterns but also reflects local peculiarities. Students faced challenges associated with remote learning, financial hardship, and disrupted social networks, compounded by gaps in digital infrastructure and institutional support (10). The World Health Organization’s announcement marking Europe’s transition to long-term COVID-19 management signaled the beginning of the post-pandemic era. Yet, COVID-19 remains a public health concern, and its lasting effects on students’ academic and personal lives require further study.

Resilience has traditionally been viewed as a stable trait, but contemporary models emphasize its dynamic and context-dependent nature, especially during large-scale disruptions such as the COVID-19 pandemic. In this study, resilience is conceptualized as a multidimensional adaptive process influenced by individual, temporal, and environmental factors consistent with Bronfenbrenner’s microsystem and chronosystem levels. In this study, resilience, which is the capacity to adapt and recover in the face of adversity, may moderate the relationship between pandemic-related stressors and mental health outcomes (20, 21). The Connor-Davidson Resilience Scale used in this study to measure resilience captures trait-like protective factors, for instance persistence and emotion regulation (22). However, resilience can also be understood as a psychological outcome a person’s demonstrated capacity to maintain or regain functioning following adversity (23). This view is reflected in Bonanno’s “Resilience Paradox,” which argues that resilience manifests across a variety of psychological profiles and does not always conform to a linear recovery model (24, 25). Therefore, resilience can be captured as both a latent disposition and a potential outcome of system-level interaction, depending on the theoretical lens applied.

Resilient students may be better able to sustain their psychological well-being despite the challenges imposed by the pandemic (26, 27). However, limited research has explored how resilience shapes mental health outcomes in the post-pandemic context, particularly within Cypriot higher education. Investigating resilience as a moderating factor offers valuable insight into protective mechanisms that can guide future interventions.

Despite the growing body of research on COVID-19 and student mental health, few studies have examined how resilience moderates the association between pandemic impact and mental health outcomes among university students in Cyprus during the post-pandemic-era.

The findings aim to inform evidence-based interventions and policy initiatives to strengthen student resilience and promote mental well-being during future crises.

## Materials and methods

### Type of study and objective

A cross-sectional study was employed to evaluate resilience and mental health post the COVID-19 Pandemic era among University Students in the Republic of Cyprus.

### Participants

The target population consisted of 30,000 full-time university students at 7 universities in 4 major districts in Cyprus, namely, Nicosia, Paphos, Limassol & Larnaca. These are the four districts in the government-controlled areas in Cyprus. Colleges and other non-university institutions were excluded to ensure consistency in academic level and institutional structure. The universities included in the study collectively represent a substantial share of the national student population, supporting the generalizability of the findings (15). The inclusion criteria were participants currently enrolled at the participating universities, at least 18 years old, and registered in Bachelors, Master’s, or PhD programs, regardless of thematic area or year of study. Purposive sampling was employed to ensure the representation of key demographic groups due to challenges in attaining proportional quotas. The sample size was determined using Kish Leslie’s formula (28).

*Infinite Population Formula:*

$$n=(Z^{2}\times p\times (1-p))/e^{2}$$

*N* = 30,000 Population size.
*Z* = 1.96 Z-score for 95% confidence.
*p* = 0.5 Population proportion.
e = 0.05 Margin of error.

The final sample consisted of 1,017 participants, exceeding the minimum required sample size.

### Instruments

The sociodemographic questionnaire was administered to assess each student’s demographic and social profile, which included for example age, gender, and relationship status. Previous research has indicated that these demographic factors might influence an individual’s capacity for resilience and mental health outcomes. To facilitate consistent analysis, key socio-demographic variables were defined as follows: age was treated as a categorical variable with four levels (18–24, 25–29, 30–34, 35+); gender was coded as a categorical variable with three levels (male, female, non-binary); and relationship status was treated as a categorical variable with three categories (Single; Married or cohabiting, Widowed or divorced). These operational definitions are consistent with national census standards and widely used approaches in mental health and public health research (13, 15, 19).

This study utilized three scales. Prior to data collection, formal approval to use each of the three instruments was granted by their respective authors.

1. The COVID-19 Impact Scale (CIS) is a self-report instrument that was used to measure the psychological effects of the COVID-19 pandemic on university students in Cyprus. It is a 10-item self-report measure assessing the psychological impact of the COVID-19 pandemic, using a 5-point Likert scale (0–4) (29). A team of academics with backgrounds in both content and scale translation oversaw the process of translating the English instrument into Greek and back into English. Under their guidance, the translation was ensured to be grammatically and culturally correct and to appropriately reflect the pandemic’s effects on the Greek population. Pilot testing was also used to establish psychometric properties (30). The CIS demonstrated strong internal consistency, with a Cronbach’s alpha of 0.96 at a 95% confidence interval. This high alpha value indicates that the scale’s items are closely related and measure the same underlying construct. The average inter-item correlation of 0.71 further contributes to the scale’s good internal consistency.
  The CIS was selected for its ability to capture residual, functional disruptions and not just acute fear or illness-related distress (29). Unlike earlier instruments such as the Fear of COVID-19 Scale (FCV-19S), which primarily measured acute health-related anxiety and emotional reactions during the initial outbreak, the CIS was designed to assess enduring functional stressors (31). Its focus on subjective functional impact over time aligns with the study’s aim to assess the persistent stressors within Bronfenbrenner’s chronosystem and exosystem domains, and its prior validation among student populations enhances its contextual relevance in 2024. Furthermore, the use of a validated questionnaire in the Greek language allowed for the accurate measurement of the psychological impact of the pandemic on the Greek university students.
2. The study assessed psychological symptomatology using the Symptom Checklist-90-Revised (SCL-90-R), a widely validated multidimensional instrument that evaluates nine primary domains of mental health, including depression, anxiety, somatization, interpersonal sensitivity, and obsessive-compulsive symptoms. Consistent with Bronfenbrenner’s microsystem, the SCL-90-R captures intrapersonal psychological functioning, offering insight into participants’ internal experiences of distress within their immediate environments. This tool was selected for its comprehensive symptom coverage, which allows for the assessment of global psychological distress as well as specific symptom clusters making it particularly suitable for detecting broad mental health impacts in the post-pandemic university student population (32, 33). Compared to narrower screening tools such as the PHQ-9 (34) or GAD-7 (35), the SCL-90-R provides greater diagnostic breadth and has been widely used in both clinical and community samples, further supporting its suitability for the current study’s multidimensional mental health focus.
  The SCL-90-R is a 90-item self-report used to assess mental health outcomes, also underwent a reliability analysis (32, 36). The analysis produced Cronbach’s alpha of 0.98 at a 95% confidence level, indicating exceptional internal consistency. The standardized Cronbach’s alpha was also 0.98, while the average inter-item correlation was 0.36. The scale has been standardized in the Greek population and has been used in other studies (37–39).
3. The Conner-Davidson Resilience Scale (CD-RISC) was used to assess the participants’ level of resilience. It is a 25-item self-report measure, assesses resilience using a 5-point Likert scale (0–4), with higher scores indicating greater resilience (22). The scale was available in Greek and had been used in Greek population (40, 41). The Cronbach’s alpha was calculated in the current study to examine its reliability. The Cronbach’s alpha was 0.96 at a 95% confidence interval, suggesting excellent internal consistency. The standardized alpha was also 0.96, demonstrating the scale’s high internal dependability which was consistent with prior reliability analysis (22, 42).
  The CD-RISC was selected for its strong psychometric properties, including high internal consistency and construct validity, as well as its broad cultural applicability and frequent use in university populations. It captures multiple facets of resilience, including tenacity, emotional regulation, personal competence, and spiritual influence, which are particularly relevant in understanding how students navigated pandemic-related disruptions (43). Framed within Bronfenbrenner’s microsystem and chronosystem, resilience was considered as both a proximal individual characteristic and a construct that unfolds over time in response to environmental stressors. Additionally, the use of the CD-RISC in the study allowed for a standardized measure of resilience, which facilitated the comparison of results with other studies that have used the same instrument to measure resilience. Additionally, Wojujutari and colleagues carried out a meta-analysis on the CD-RISC scales adaptation including the Greek version they found that there was no significant moderation by language showing that its psychometric properties are robust across translations (44).
  Alternative resilience measures were reviewed but were not selected due to theoretical and practical limitations. The Brief Resilience Scale (BRS) (45) offers a unidimensional assessment focused narrowly on the ability to recover from stress, lacking the multifactorial depth needed for this study’s mediation and moderation analyzes. The Resilience Scale for Adults (RSA), while comprehensive, is relatively long and more suited to clinical assessment contexts, raising concerns about respondent fatigue in large-scale student surveys (46). In contrast, the CD-RISC provides an optimal balance of conceptual richness, brevity, and empirical rigor, aligning closely with the study’s aim to examine resilience as a potential moderator of the relationship between COVID-19 impact and mental health outcomes.

### Procedure

**Figure 1:**
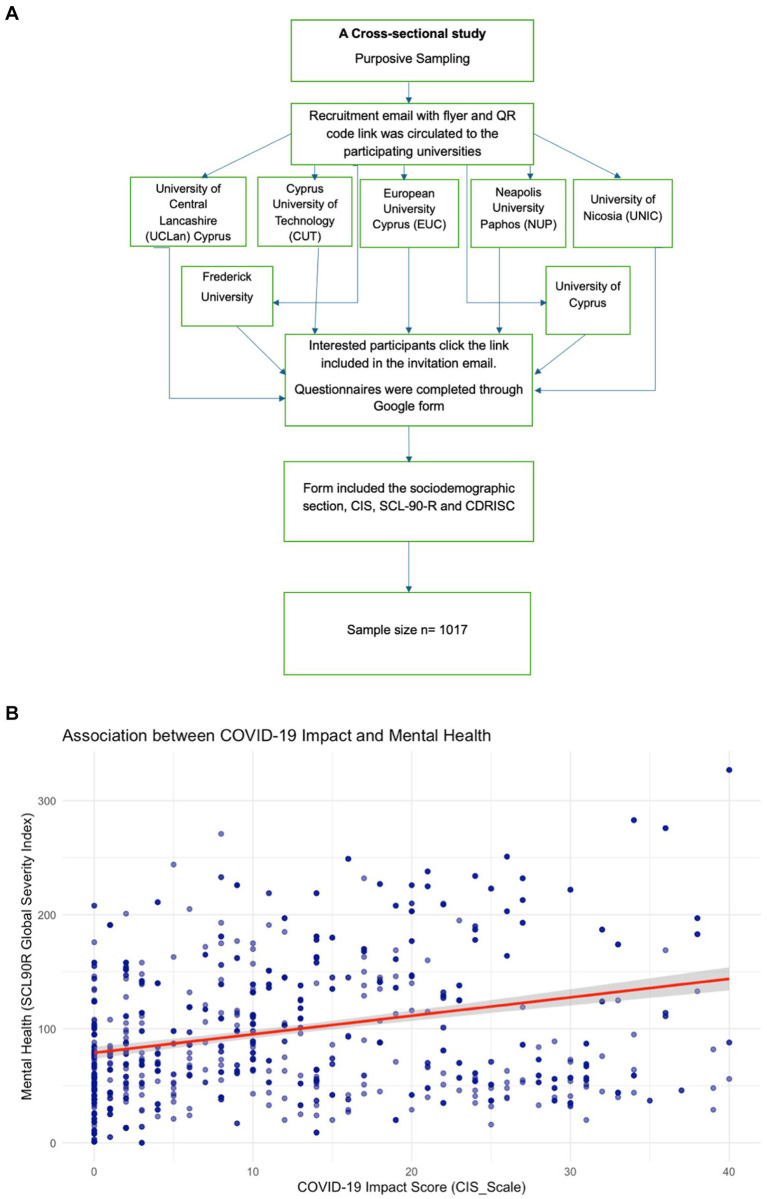
**(A)** Flowchart of the study design and data collection. **(B)** Scatter plot showing the relationship between Covid-19 impact and mental health. The line represents the linear regression fit.

Data was collected on a web-based questionnaire using Google Forms through a link that was shared to students via email. The form included the sociodemographic section, CIS, SCL-90-R and CDRISC, which were available to participants in both Greek and English. The participating universities sent all their registered students an email explaining the study, including the survey link. As illustrated in Figure 1A. This email informed participants about the research objectives, the use of their data in line with the General Data Protection Regulation and provided the Principal Investigator’s contact details for any additional questions. The google forms were secured and optimized for data collection by ensuring that duplicate entries were prevented and only authorized users from the participating universities could respond using Google Forms. Response editing was disabled, in addition access to data was restricted, and HTTPS encryption secured data transmission. Input validation and real-time monitoring ensured data integrity and security.

### Ethical considerations

Interested individuals provided digital informed consent before completing the approximately 25 min questionnaire. Participation was anonymous, voluntary and participants could withdraw at any time and for any reason. The study was approved by the Cyprus Bioethics Committee (approval no: EEC/EP/2023/31).

### Data analysis

Statistical analyzes were performed using R version v4.5.1 for Mac (47). Descriptive analyzes were conducted to summarize the numeric variables of interest, which included mean, median, standard deviation (s.dev), and interquartile range (IQR), to describe all numeric variables, depending on the distribution of each variable. Categorical variables were described using absolute frequencies and proportions. The study employed linear regression analysis to investigate the main associations of interest addressing the study aims. For Aim 1, we analyzed the association between COVID-19 impact (numeric independent variable) and mental health outcomes (numeric dependent variable), using linear regression adjusting for age, gender, marital status, education, university, socioeconomic status, funding type, and religious attendance. Aim 2 examined the relationship between COVID-19 impact (numeric independent variable) and resilience (numeric dependent variable), using a multiple linear regression with similar adjustments for confounders. For Aim 3, investigated whether resilience (numeric independent variable) predicted mental health outcomes (numeric dependent variable), using multiple linear regression while controlling for age, gender, marital status, educational level, university, socioeconomic status, funding type, religious attendance frequency, and academic discipline. Aim 4, we assessed the moderating effect of resilience on the association between COVID-19 impact and mental health symptoms, using simple slopes controlling for the aforementioned confounders.

## Results

### Demographic characteristics of the sample

The study sample consisted of 1,017 participants from seven universities. Table 1 presents the sociodemographic characteristics of the sample. The majority of participants were from European University Cyprus 403 (39.6%), Neapolis University Pafos 208 (20.5%), University of Nicosia 161 (15.8%), Cyprus University of Technology 125 (12.3%), University of Central Lancashire 57 (5.6%), University of Cyprus 32 (3.2%) and Frederick University 30 (3.0%). In terms of age distribution, 61.2% were 18–24 years old, 25.0% were 25–29, 6.5% were 30–34, and 7.3% were 35 or older. The gender distribution comprised 338 (33.2%) men, 576 (56.6%) women, 88 (8.6%) non-binary individuals, and 15 (1.4%) who identified as other. Participants reported their marital status, with 448 (44.0%) being married or cohabiting, 556 (54.6%) single, and 11 widowed or divorced (1.0%), while two (0.2%) declined to disclose. The majority were pursuing bachelor’s degrees. Religious service attendance varied, with 334 (32.8%) attending once, 237 (23.3%) twice, 174(17.1%) three times, and 272 (26.7%) four times per month, indicating the highest number attended once monthly and the lowest three times.

**Table 1:** Demographic characteristics of the participants.

Age	Mean 23.81 (SD 4.90)
Age-group *n* (%)	
18–24	623 (61.2%)
25–29	254 (25.0%)
30–34	66 (6.5%)
35+	74 (7.3%)
Gender *n* (%)	
Women	576 (56.6%)
Men	338 (33.2%)
Non-binary	88 (8.6%)
Other	15 (1.4%)
Marital status *n* (%)	
Single	556 (54.6%)
Married or cohabiting	448 (44.0%)
Widowed or divorced	11 (1.08%)
University *n* (%)	
European University Cyprus	403 (39.6%)
Neapolis University Pafos	208 (20.4%)
University of Nicosia	161 (15.8%)
Cyprus University of Technology	125 (12.2%)
University of Central Lancashire	57 (5.6%)
University of Cyprus	32 (3.1%)
Frederick University	30 (2.9%),
Other	1 (0.10%)
Educational level *n* (%)	
Bachelors	741 (72.8%)
Masters	245 (24.1%)
PhD	28 (2.8%)
Other	3 (0.3%)
Discipline *n* (%)	
Business, finance and communication	170 (16.72%)
Health and Life Sciences	426 (41.89%)
Social sciences and education	116 (11.41%)
Engineering and IT	143 (14.06%)
Law and politics	109 (10.72%)
Other	53 (5.21%)
Education funding	
Family	674 (66.3%)
Scholarship	64 (6.3%)
Self/loan	267 (26.3%)
Missing	12 (1.2%)
Religious service attendance per month	
Once	334 (32.8%)
Twice	237 (23.3%)
Three times	174 (17.1%)
Four times	272 (26.7%)

Table 2 displays descriptive statistics for the variables of interest in this study. The mean score on the Covid Impact scale (CIS Scale) was 11.59 (SD = 10.73), with a positive skew (0.71), indicating that most participants scored on the lower end of the scale. The mental health symptoms checklist 90 revised (SCL-90-R) with values ranging from 0 to 327, the SCL-90-R total score had a moderate right skew (0.85) and a mean of 97.79 (SD = 60.64). The Connor-Davidson Resilience Scale (CDRISC) showed a small negative skewness (−0.42) and an average score of 55.82 (SD = 21.12), suggesting that greater resilience scores were somewhat more prevalent in the group.

**Table 2:** Descriptive statistics of the study sample.

Instrument	Mean	SD	Skewness	Min	Max
CIS Scale	11.58	10.731	0.70	0	40
SCL90R_scale	97.78	60.64	0.84	0	327
SCL90R_Somatization	12.60	9.34	0.94	0	48
SCL90R_Obsessive Compulsive	13.23	7.59	0.39	0	34
SCL90R_Interpersonal	10.27	6.27	0.73	0	30
SCL90R_Depression	15.59	9.79	0.64	0	44
SCL90R_Anxiety Scale	10.81	8.01	0.90	0	40
SCL90R_Anger Hostility	6.29	5.05	0.93	0	23
SCL90R_Phobic Anxiety	5.62	5.41	1.13	0	28
SCL90R_Paranoid Ideation	7.22	4.86	0.67	0	24
SCL90R_Psychoticism	8.61	7.24	1.19	0	37
CDRISC_scale	55.82	21.12	−0.42	0	99

Descriptive statistics of the study sample for the Covid Impact Scale (CIS) total score, the Symptom Checklist-90-Revised (SCL-90-R) and its subscales (Somatization, Obsessive-Compulsive, Interpersonal Sensitivity, Depression, Anxiety, Anger Hostility, Phobic Anxiety, Paranoid Ideation, Psychoticism), and the Connor–Davidson Resilience Scale (CD-RISC) total score. Values presented include the mean, standard deviation (SD), skewness, and observed minimum and maximum scores.

### Main associations of interest

In order to address the first study, aim, that is to determine the association between the COVID-19 pandemic impact and mental health outcomes among university students during the post-pandemic era, in the Republic of Cyprus, a scatterplot was initially constructed, followed by linear regression analysis as illustrated in Figure 1B.

A linear regression analysis was performed while adjusting for several potential confounders such as age, gender, marital status, education level, university, socioeconomic status, funding type, and frequency of religious attendance. The model revealed a significant positive association between COVID-19 Impact and mental health symptoms *β* = 2.54, *p* < 0.001, 95% CI (2.17, 2.91). For every unit increase in COVID-19 impact, the model predicted a 2.54 increase in mental health symptoms. The positive regression coefficient suggests a direct relationship, where higher scores on the COVID-19 Impact Scale are associated with higher mental health symptoms severity. Although the model for Aim 1 showed a statistically significant relationship, the proportion of variance explained was low. This suggests that while the COVID-19 pandemic and its mitigation measures had an impact on mental health symptoms, they only accounted for a small portion of the overall variability.

**Figure 2:**
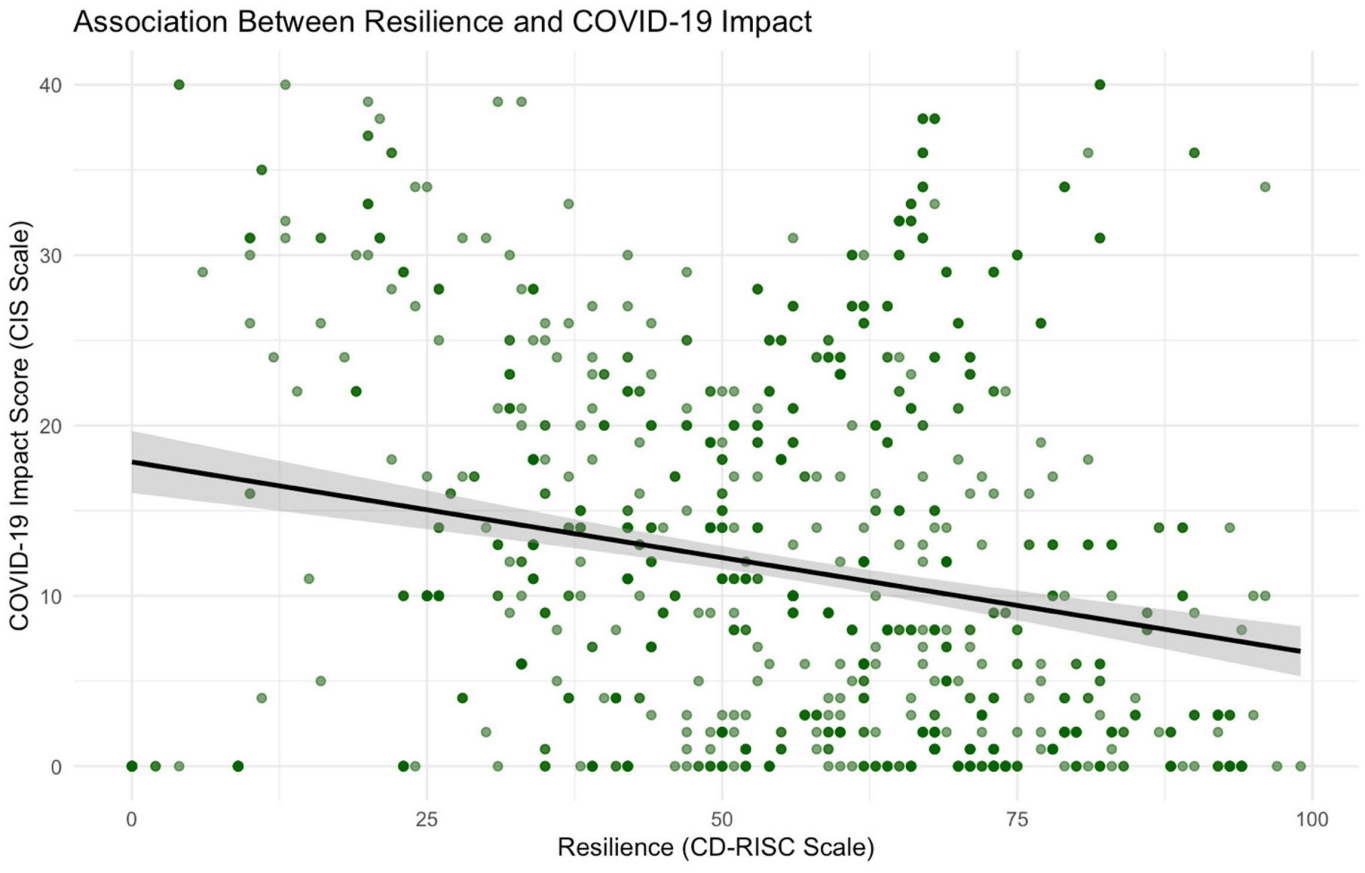
Scatter plot showing the relationship between resilience and Covid-19 impact. The line represents the linear regression fit.

A multiple linear regression was conducted to examine the association between resilience and COVID-19 Impact (aim 2) as illustrated in Figure 2, adjusting for confounders including age, gender, marital status, education level, university, socioeconomic status (SES), funding type, and religious attendance frequency. The analysis revealed a significant association between resilience and COVID-19 impact *β* = −0.08, *p* < 0.001, CI (−0.11, −0.06), indicating that higher resilience scores were associated with lower COVID-19 Impact. This indicates that for each unit increase in resilience, there was a 0.08-point decrease in COVID-19 Impact, holding all other variables constant.

A multiple linear regression analysis examined whether resilience was associated with. Mental health symptoms as illustrated in Supplementary Figure 1. The analysis controlled for age, gender, marital status, educational level, university, socioeconomic status, funding type, religious attendance frequency, and academic discipline. Results indicated that resilience significantly and negatively predicted mental health symptoms *β* = −0.31, *p* < 0.001, 95% CI (−0.48, −0.14) suggesting that higher resilience was associated with fewer mental health symptoms.

In addition, a significant interaction was found between COVID-19 impact and resilience *β* = 1.51, *p* < 0.001, CI (1.00, 2.03). The simple slopes analysis was conducted to examine the moderating effect of resilience on the association between COVID-19 impact and mental health symptoms. Comparing the university students categorized as having low versus high resilience.

**Figure 3:**
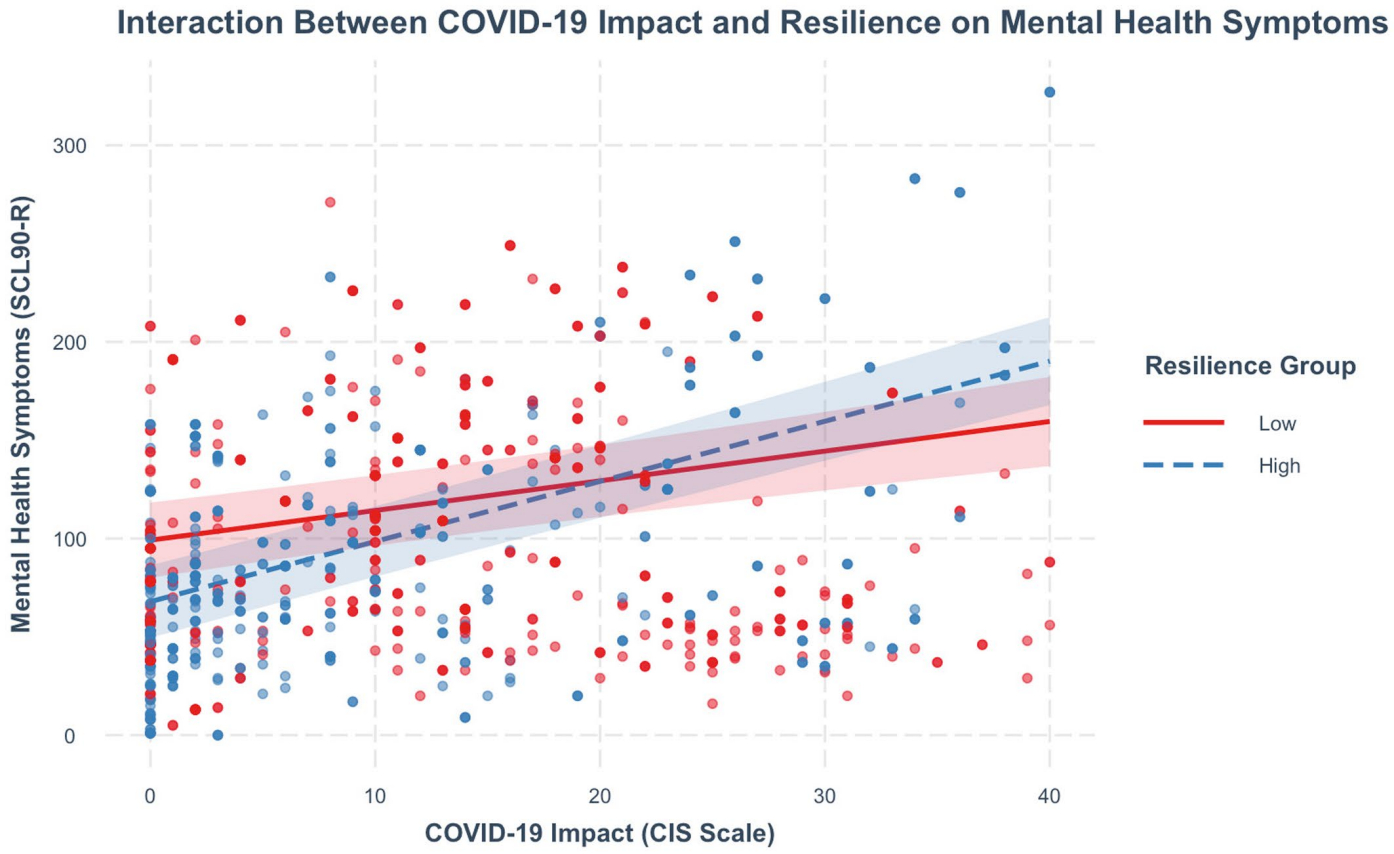
Simple slopes indicating the interaction between COVID 19 impact and resilience on mental health symptoms.

Among the university students with low resilience, the relationship between COVID-19 Impact and mental health symptoms was statistically significant (*β* = 1.52, SE = 0.26, t = 5.80, *p* < 0.001). Among those classified as having high resilience, the association was even stronger (*β* = 3.09, SE = 0.22, t = 13.76, *p* < 0.001). These results indicate that the effect of Covid Impact on mental health was significant only among individuals with high resilience as illustrated in Figure 3.

## Discussion

### Summary of findings

Our findings highlight the continued psychological impact of COVID-19 on university students in the post-pandemic era. The integration of the Bronfenbrenner’s ecological framework in this study demonstrates that mental health outcomes are shaped by an interplay of individual and systemic factors. Resilience emerged as a key moderator, with higher levels associated with lower mental health symptom burden across contexts. These results advance understanding of resilience as a context-sensitive process rather than a fixed trait and underscore the relevance of examining psychological adaptation through a multilevel ecological lens.

### Participant demographics and contextual considerations

This study was conducted within a university population primarily comprising single individuals, with a majority identifying as female and most falling within the 18 to 24 age range. Although the sample also included males, non-binary participants, and individuals from other age groups, the predominance of this demographic likely influenced the patterns observed. This age group is typically characterized by significant academic, social, and developmental transitions, which may shape their perceptions and behaviors. Consequently, while the findings provide valuable insights into the experiences of this dominant subgroup, they should be interpreted within the broader context of the study’s diverse participant pool in the same environment.

Against this demographic backdrop, the study’s findings show important patterns aligned with the four aims, which are discussed below in relation to their contextual and theoretical implications.

### Depression trajectories post-pandemic

Aligned with the first aim of the study, findings revealed a positive association between the impact of the COVID-19 pandemic and mental health symptoms among university students in the Republic of Cyprus during the post-pandemic period. Specifically, for every unit increase in the COVID-19 Impact Scale, there was a corresponding 2.54 unit increase in mental health symptoms as measured by the SCL-90-R. This result aligns with existing literature that has documented the widespread psychological consequences of the pandemic (48, 49). Additionally, numerous previous studies have demonstrated that the COVID-19 pandemic had a significant detrimental effect on university students’ mental health, with anxiety, depression, and stress being the most common psychological challenges experienced (50, 51). The prolonged lockdowns resulted in social isolation, which damaged essential peer relationships and support structures, leading to reduced emotional outlets and heightened feelings of loneliness and alienation among students (48, 52).

Other unmeasured factors such as pre-existing mental health conditions, cultural perspectives on mental health, and individual coping strategies potentially played a role in shaping these outcomes (50, 53). These results reinforce the urgent need for accessible mental health services and preventative interventions for university students in post-crisis contexts.

### Resilience as a protective factor

The second aim of the study explored the association between resilience and COVID-19 Impact. Results indicated that higher resilience scores were associated with lower COVID-19 Impact. This finding aligns with prior research demonstrating the buffering role of resilience in times of adversity (54, 55). For example, Verdolini and colleagues, reported that individuals with higher resilience were better equipped to manage pandemic-related stressors (56). Resilience supports adaptive functioning and psychological recovery during crises, including public health emergencies (30, 57). Individuals with greater resilience were better able to manage anxiety and stress during the pandemic, underscoring the protective role of resilience in times of crisis (56, 58). These findings underscore the potential of resilience-building programs such as stress management workshops, cognitive behavioral strategies, and peer support groups as preventative tools to bolster student mental health in uncertain times (59, 60).

### Unexpected patterns in resilience’s moderating role

Simple slopes analysis further revealed a moderating effect of resilience on the relationship between COVID-19 Impact and mental health symptoms. Interestingly, the association between COVID-19 Impact and mental health symptoms was stronger among individuals with higher resilience compared to those with lower resilience. This finding diverges from common assumptions that high resilience universally mitigates negative outcomes. One explanation may be that even highly resilient individuals can become overwhelmed when stress is sustained over long periods. As suggested by Celbis and peers, early resilience may erode over time, leading to burnout (61), and those who showed resilience early on may suffer from burnout, which will eventually impair their resilience. Xu and colleagues further argue that those high in resilience may experience less post-traumatic growth because they are less emotionally reactive to adverse events (62). Additionally, the burden of continued coping may become unsustainable, particularly in protracted crises like the COVID-19 pandemic.

These findings suggest that resilience may not function in a strictly linear fashion and may be influenced by contextual, cultural, and temporal factors. For instance, the resilience scale used in this study may not fully capture the unique experiences of Cypriot university students. Moderating variables such as access to mental health resources, availability of social support, and individual coping strategies may further complicate this relationship. It’s also plausible that students with low resilience had already reached a ceiling in mental health symptoms, leaving little room for COVID-19 Impact to increase those symptoms further (63, 64).

### Trends in student well-being over time

This study’s results point to ongoing concerns about the mental health of university students even after the height of the COVID-19 crisis. These patterns suggest that the effects of the pandemic persist beyond the immediate aftermath and may evolve over time. Without appropriate interventions, post-crisis psychological effects may accumulate and interfere with students’ academic, social, and developmental outcomes. This highlights the necessity for ongoing mental health support tailored to the unique needs of university populations, especially during societal recovery periods.

### Study limitations

Several limitations should be considered when interpreting these results. The study employed a cross-sectional design, limiting the ability to determine causal or directional relationships. Additionally, the use of self-report measures introduces potential for bias, such as under- or over-reporting due to social desirability or recall error. Future studies using longitudinal designs and culturally adapted tools are recommended to deepen understanding and improve generalizability. While this study measured resilience using a validated trait-based instrument (CD-RISC), it is important to recognize that resilience may also function as an outcome variable that develops over time or emerges in response to specific contextual factors (65).

Although the COVID-19 Impact Scale was selected for its ability to capture residual functional disruptions rather than acute fear it remains a self-report measure limited to a single time point (29). Although the CIS captures broader, subjective impacts aligned with the macrosystem and exosystem, its cross-sectional administration in this study restricts the ability to assess how COVID-related stressors fluctuate or accumulate over time.

### Implications for practice

The findings from this study underscore the vital importance for institutions to have comprehensive and multidimensional mental health support systems. Universities can leverage these insights to develop and implement evidence-based interventions that address the specific challenges faced by students. Some examples of how this can be achieved include establishing peer support networks (66) to enhance social connectivity and reduce stigma, introducing stress management seminars grounded in cognitive behavioral techniques, and implementing resilience training programs that equip students with flexible coping strategies with a focus on students facing socioeconomic hardships and/or limited social support (67, 68). At the policy level, the results emphasize the need for greater investment in mental health resources and services, both within universities and in the broader community. Additional research investigation on these matters should ideally utilize longitudinal methodologies and involve wider population groups, enhancing a more holistic understanding of the complex interplay of factors influencing student mental health outcomes. Longitudinal designs could potentially capture the evolving nature of resilience and distress over time. Incorporating models such as Bonanno’s Resilience Paradox may illuminate the diversity of adaptation trajectories beyond traditional recovery frameworks. Furthermore, mental health strategies must extend beyond individual coping to encompass structural supports such as academic accommodations, community-based engagement, and spiritual or peer networks. Embedding psychological support within students’ lived environments may help buffer ongoing stress exposure and foster long-term recovery. Overall, this study lays the groundwork for empirical and policy efforts that address both the immediate and lasting mental health effects of global disruptions among emerging adult populations.

## Conclusion

The mental health of university students in the Republic of Cyprus was greatly affected by the COVID-19 pandemic, while higher resilience was associated with lower COVID-19 Impact and mental health burden. To potentially lessen the pandemic’s long-term effects, public health authorities should concentrate on finding efficient interventions that support mental health and resilience. Research examining resilience’s mediation function in the connection between stressors linked to major crises and their role in mental health may yield important new vital information. The creation of focused interventions may also be influenced by research into pre-existing protective qualities, such as psychological wellbeing.

## Data Availability

The raw data supporting the conclusions of this article will be made available by the authors, without undue reservation.
