# Supplementary material for: Resilience and mental health in university students post-COVID-19 pandemic: insights from the Republic of Cyprus
Source: Front Public Health. 2025 Sep 1;13:1638427. doi: 10.3389/fpubh.2025.1638427 (PMC12433969; doi:10.3389/fpubh.2025.1638427)
Supplement: Supplementary file 1 [file Supplementary_file_1.docx]

Supplementary Table S1 Descriptive statistics of the study sample

|  | Mean | SD | Skewness | Min | Max |
| --- | --- | --- | --- | --- | --- |
| CIS Scale | 11.58 | 10.731 | 0.70 | 0 | 40 |
| SCL90R_scale | 97.78 | 60.64 | 0.84 | 0 | 327 |
| SCL90R_Somatization | 12.60 | 9.34 | 0.94 | 0 | 48 |
| SCL90R_Obsessive Compulsive | 13.23 | 7.59 | 0.39 | 0 | 34 |
| SCL90R_Interpersonal | 10.27 | 6.27 | 0.73 | 0 | 30 |
| SCL90R_Depression | 15.59 | 9.79 | 0.64 | 0 | 44 |
| SCL90R_Anxiety  Scale | 10.81 | 8.01 | 0.90 | 0 | 40 |
| SCL90R_Anger Hostility | 6.29 | 5.05 | 0.93 | 0 | 23 |
| SCL90R_Phobic  Anxiety | 5.62 | 5.41 | 1.13 | 0 | 28 |
| SCL90R_Paranoid  Ideation | 7.22 | 4.86 | 0.67 | 0 | 24 |
| SCL90R_Psychoticism | 8.61 | 7.24 | 1.19 | 0 | 37 |
| CDRISC_scale | 55.82 | 21.12 | -0.42 | 0 | 99 |


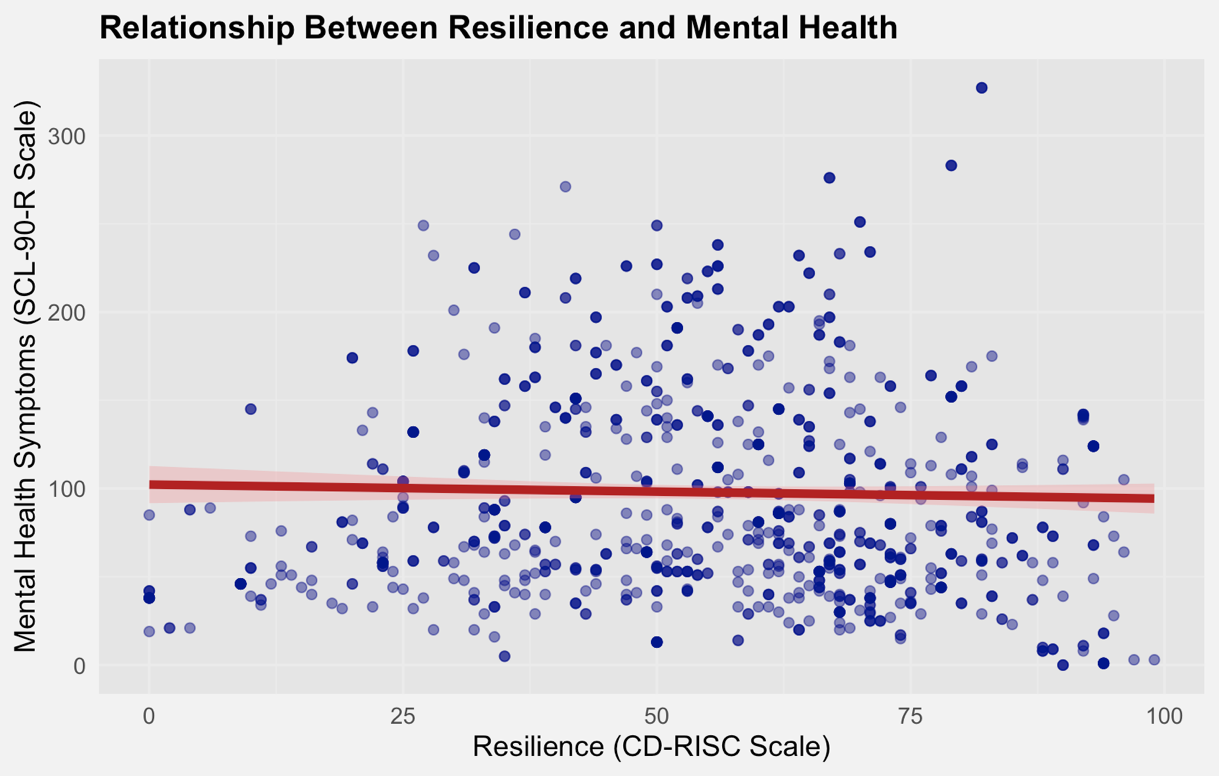


Supplemental Figure 1. Scatter plot illustrates the relationship between Resilience and Mental Health. The regression line indicates no significant association between the variables.
